# Biosynthesis of Stable Antioxidant ZnO Nanoparticles by *Pseudomonas aeruginosa* Rhamnolipids

**DOI:** 10.1371/journal.pone.0106937

**Published:** 2014-09-04

**Authors:** Brahma Nand Singh, Ajay Kumar Singh Rawat, Wasi Khan, Alim H. Naqvi, Braj Raj Singh

**Affiliations:** 1 Pharmacognosy and Ethnopharmacology Division, CSIR-National Botanical Research Institute, Lucknow, Uttar Pradesh, India; 2 Centre of Excellence in Materials Science (Nanomaterials), Department of Applied Physics, Z. H. College of Engineering and Technology, Aligarh Muslim University, Aligarh, Uttar Pradesh, India; RMIT University, Australia

## Abstract

During the last several years, various chemical methods have been used for synthesis of a variety of metal nanoparticles. Most of these methods pose severe environmental problems and biological risks; therefore the present study reports a biological route for synthesis of zinc oxide nanoparticles using *Pseudomonas aeruginosa* rhamnolipids (RLs) (denoted as RL@ZnO) and their antioxidant property. Formation of stable RL@ZnO nanoparticles gave mostly spherical particles with a particle size ranging from 35 to 80 nm. The RL@ZnO nanoparticles were characterized by UV-visible (UV–vis) spectroscopy, scanning electron microscopy, transmission electron microscopy, dynamic light scattering, Fourier transform infrared spectroscopy, X-ray diffraction (XRD), and thermal gravimetric analysis. The UV–vis spectra presented a characteristic absorbance peak at ∼360 nm for synthesized RL@ZnO nanoparticles. The XRD spectrum showed that RL@ZnO nanoparticles are crystalline in nature and have typical wurtzite type polycrystals. Antioxidant potential of RL@ZnO nanoparticles was assessed through 2,2–diphenyl-1-picrylhydrazyl (DPPH), hydroxyl, and superoxide anion free radicals with varying concentration and time of the storage up to 15 months, while it was found to decline in bare ZnO nanoparticles. Similarly, the inhibitory effects on β-carotene oxidation and lipid peroxidation were also observed. These results elucidate the significance of *P*. *aeruginosa* RL as effective stabilizing agents to develop surface protective ZnO nanoparticles, which can be used as promising antioxidants in biological system.

## Introduction

In recent years, semiconductor zinc oxide (ZnO) has gained momentum due to their unique properties such as electronic, structural and thermal [1]. It has been used considerably for its important applications in different areas viz. catalysts [2], sensors [3], optoelectron, highly functional, and effective photoelectron devices [4]. ZnO nanostructures have a great advantage to apply in medical and pharmaceutical applications due to their large surface area and high catalytic activity [1]. The ZnO is widely used in baby powder, calamine cream, anti-dandruff shampoos, and antiseptic ointments as a potential antimicrobial agent [5].

In recent years, various methods have developed to synthesize ZnO nanoparticles, however several glitches are reported with these methods related to stability of the nanoparticles, control of the crystal growth, aggregation of the particles, and heterogenecity resulting in broadening of the particle size of distributions [6]. The ‘biosynthesis’ is an environmentally friendly process in chemistry and chemical technology is becoming increasingly popular and is much needed as a result of worldwide problems associated with environmental contamination [7], [8]. Products from nature such as microbial surfactants have been used as reductants and as capping agents during synthesis [6], [9], [10]. Very recently, we have demonstrated that the application of *Bacillus subtilis* bio-surfactants a template for synthesis of cadmium sulfate quantum dots and as a surface modifying agent for enhancement of their stability at room temperature [9]. Rhamnolipids (RLs) are cyclic lipopeptide surfactants with molecular mass usually from 500 to 1500 Da, and critical micelle concentration in the range from 1 to 200 mg/L [6], [11]. The focus of this study was to develop one-pot method for biosynthesis of highly stabilized ZnO nanoparticles using *Pseudomonas aeruginosa* RLs.

Evaluation of pharmacological properties of nanomaterials has become one of the significant basic studies in biomedical sciences. ZnO nanoparticles have aroused considerable interest recently because of their potential beneficial effects on human health-they have been reported to have strong anticancer [12], antimicrobial [13], and antioxidant activities [14]. Antioxidants are compounds that protect cells against the damaging effects of reactive oxygen species (ROS) [15], [16], [17]. An imbalance between antioxidants and ROS results in oxidative stress, leads to cellular damage [18], [19], [20]. Very recently, Das and colleagues partially examined the antioxidant activity of ZnO nanoparticles using 1,1-diphenyl-2-picryl-hydrazyl (DPPH) free radical scavenging assay [14]. However, DPPH radical scavenging activity is not enough to claim the antioxidant potential of these nanomaterials. Additional experiments are required to claim their antioxidant activity. There is a great need for the evaluation of antioxidant potential of ZnO nanoparticles in detail. In this study, we examined the comparative antioxidant activity of RL-stabilized (denoted as RL@ZnO) and bare ZnO nanoparticles using standard *in-vitro* antioxidant activity assays including reducing power, DPPH, hydroxyl (^•^OH) and superoxide anion (O_2_^•−^) free radicals scavenging, β-carotene bleaching, and lipid peroxidation etc.

## Results and Discussion

A range of culturable microbes are known to produce surface active bio-surfactants with distinct advantages over the chemical polymers in terms of high specificity, biodegradability, biocompatibility, and solubility enhancement and therefore regarded as an environmentally friendly approach [11], [21], [22], [23]. Moreover, bacterial RLs have recently been used for capping, stabilizing and dispersant of nanomaterials [9], [24]. Therefore, bio-prospecting of RL producing bacteria have gained much interest due to their industrial applications. Although, there is a report on RL-mediated surface modification of ZnO nanoparticles, however there is no report on comparative studies of RL@ZnO and bare ZnO nanoparticles to date with respect to their antioxidant activity. Considering these points, in this investigation, we report evidence for RL@ZnO nanoparticles in relation to their antioxidant activity. Toward this end, RLs were isolated from *P*. *aeruginosa* CEMS077 and used directly for synthesizing and stabilizing agents in the reaction mixture during the fabrication of antioxidant nanoparticles.

### Molecular characterization of RL producing CEMS077

**Figure 1:**
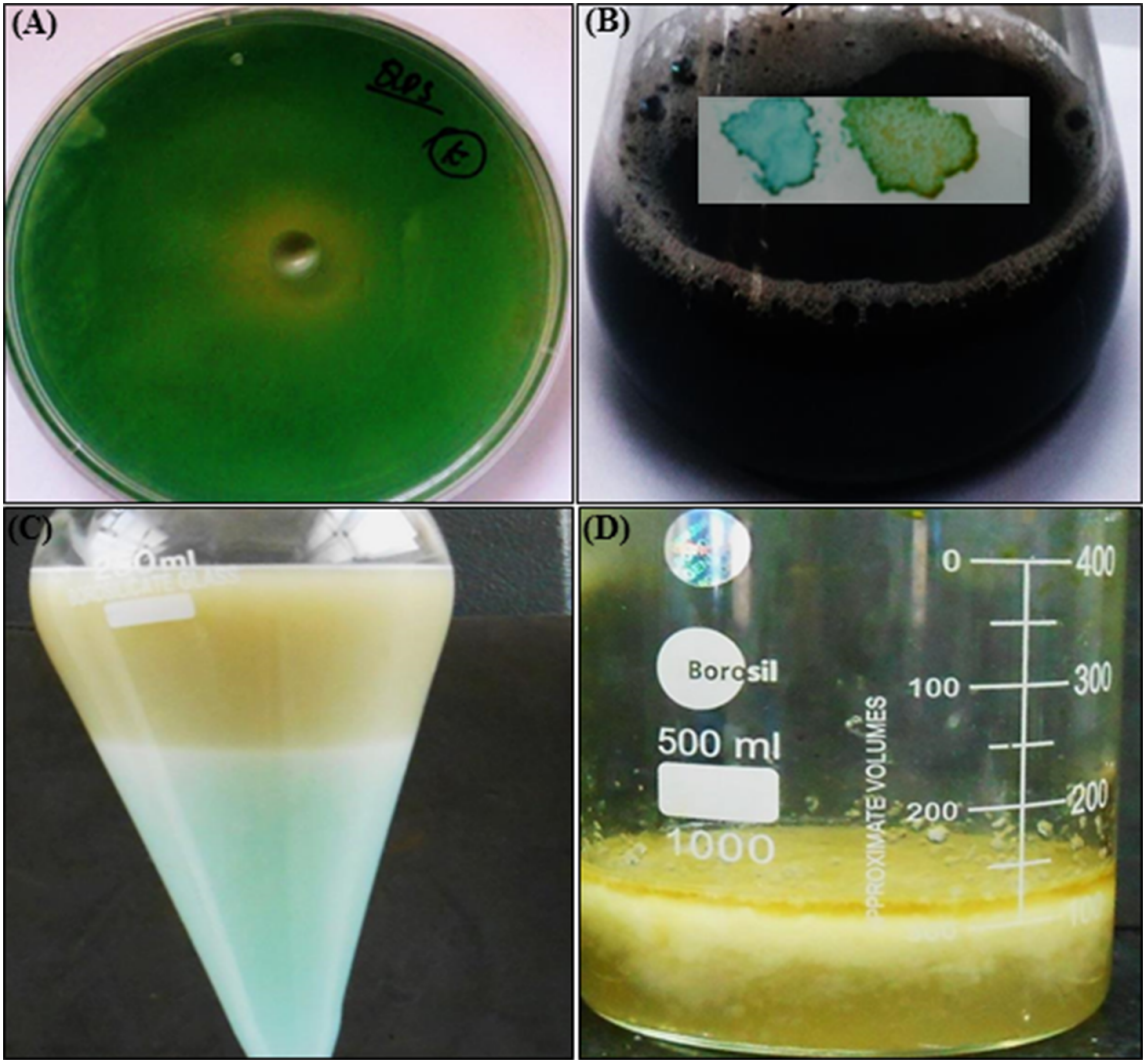
RL production by *P. aeruginosa* CEMS077. Production on nutrient agar (A) and in nutrient broth medium (B inset) validated by using methylene blue and cetyl trimethylammonium bromide (CTAB) method. (C) Extraction and isolation of RLs. To promote the RL production, nutrient broth was supplemented by 3% glycerol and the pH of medium was adjusted to 7.0±0.2. The sterilized medium was inoculated with 0.5% freshly overnight grown culture of CEMS077 and incubated at 30±1°C under static condition for 96 h. Then, supernatant was collected through centrifugation and acidified (12N HCl) to precipitate RLs. The mixture of chloroform-ethanol (2∶1) was used to extract RLs and solvents were evaporated under reduced pressure. (D) Extracted RLs along with methanol.

The CEMS077 showed RL production on agar (Figure 1A) and broth (Figure 1B) media. The yield of RLs was determined to be 329 mg/L (Figure 1C and D) and characterized by HPLC and HPTLC (Figure S1A and S1B in File S1). The chemical structure of the most abundant component was identified as RL-C_10_-C_12∶1_ (4.5%), RL-C_8_-C_10_ (2.5%), RL-C_10_-C_10_ (51.1%), RL-RL-C_8_-C_10_ (1.8%), RL-RL-C_10_-C_14:1_ (30.9%), and RL-C_10_-C_12_ (9.2%).

**Figure 2:**
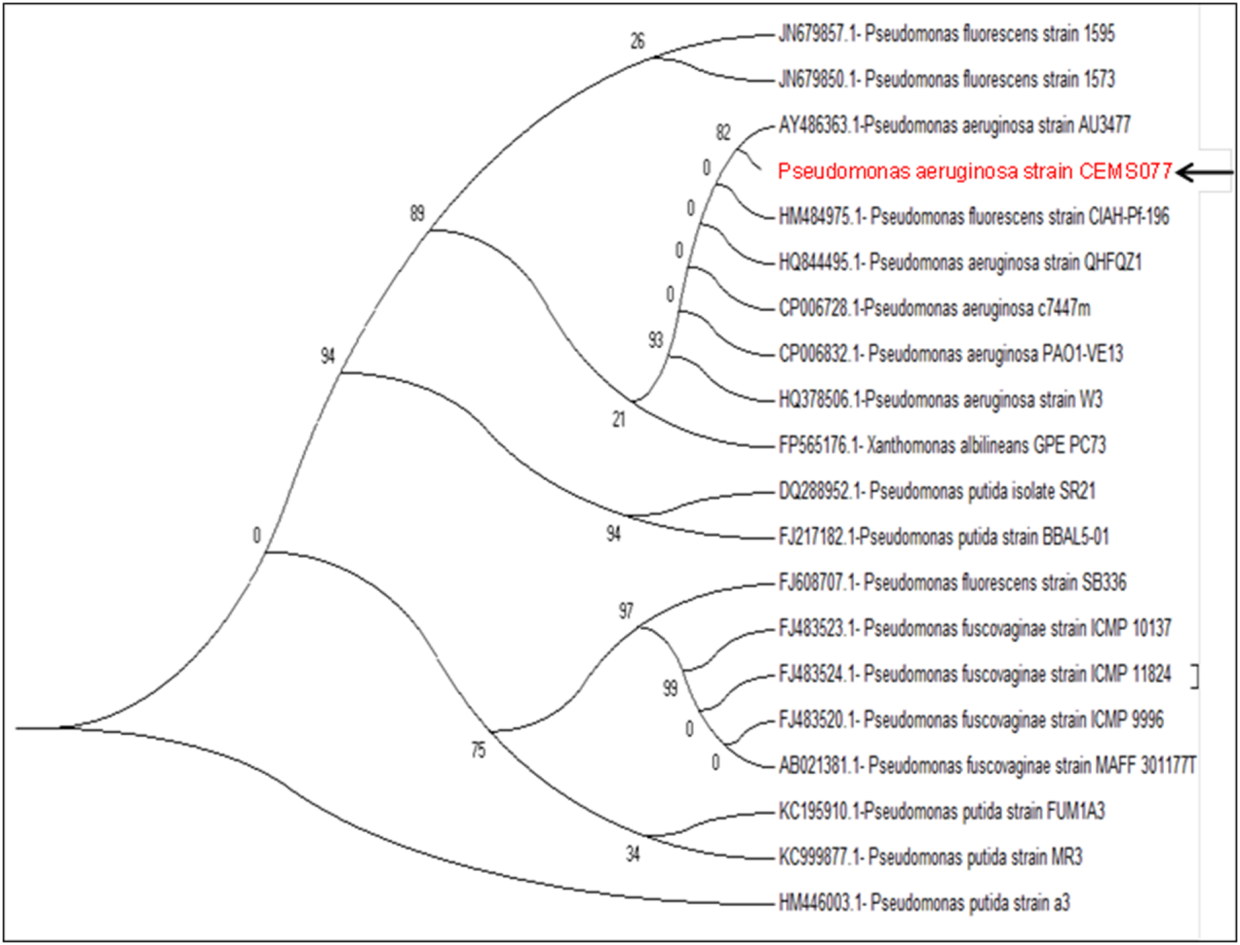
Molecular characterization of *P*. *aeruginosa* CEMS077. Phylogenetic tree showing relationship of CEMS077 with other strains of *Pseudomonas* species based on 16S rRNA gene sequences retrieved from NCBI GenBank. Arrow represents the status of strain CEMS077 from this study in phylogenetic tree.

The CEMS077 was identified and characterized based on the phylogenetic analyses of gene sequences of 16S rRNA. The PCR amplification and sequencing yielded the nucleotide sequences of 1.5 kbp for 16S rRNA gene. The Blastn sequence alignment analysis revealed that the ∼99% sequence homology of 16S rRNA gene with the strains of *P*. *aeruginosa*. The phylogenetic analysis revealed the close relationship of CEMS077 with the complete genome sequenced of *P*. *aeruginosa* strains (Figure 2).

### Biosynthesis mechanism of nanoparticles

**Figure 3:**
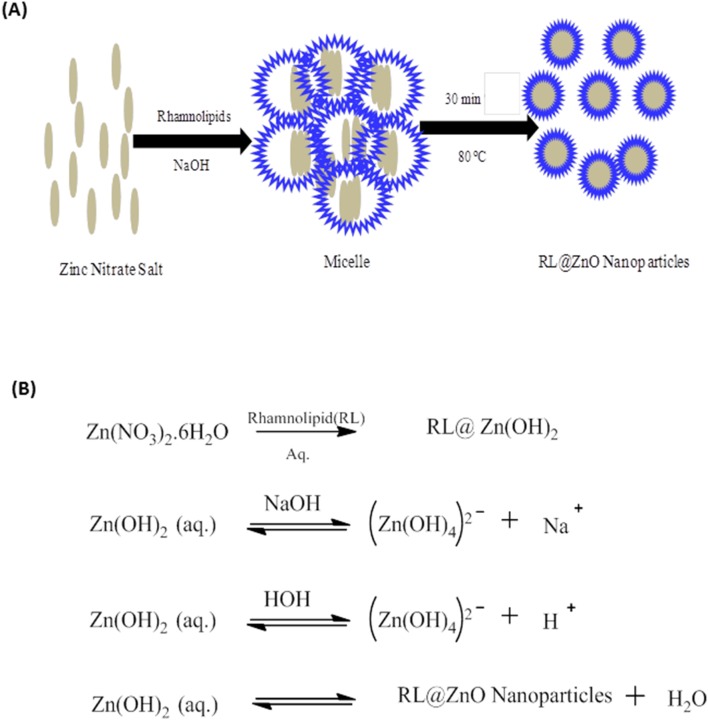
Biosynthesis mechanism of RL@ZnO nanoparticles. (A) The schematic representation of biosynthesis of RL@ZnO nanoparticles and (B) Chemical reactions involved in synthesis of the RL@ZnO nanoparticles by sol–gel method.

A possible mechanism for synthesis of the RL@ZnO nanoparticles is suggested as follows. RLs in aqueous solution disperse in the form of spherical core–shell micellar structure in which the core is represented the hydrophobic C–C chains and the shell consist of polar –OH groups. The RLs molecule hydrophobic alkyl chains get attached to the surface of primary ZnO crystallite in the presence of oxygen. The high surface energy of pre-formed ZnO crystallite begins to RL@ZnO nanoparticles. Synthesis of the ZnO nanoparticles proceeds inside the core of the small micelles of RLs *via* the nucleation and growth of ZnO nanoparticles (Figure 3A). The first step includes the formation of zinc hydroxide upon the addition of NaOH with zinc nitrate aqueous solution and crystalline ZnO nucleate from the dehydration of preformed ZnOH. The plausible synthesis reaction is elucidated in Figure 3B. Numerous studies have been proposed the similar mechanistic conclusions [25].

### Characterization of RL@ZnO nanoparticles

**Figure 4:**
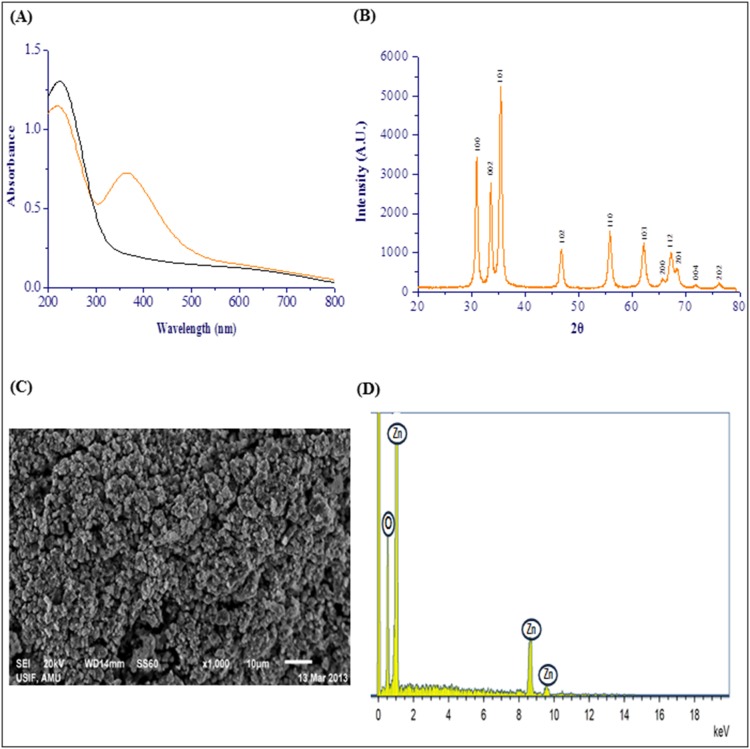
Characterization of RL@ZnO nanoparticles. (A) UV–vis spectra of RL@ZnO nanoparticles (orange line) and RL (black line). A characteristic absorption peak of RL@ZnO nanoparticles (10 µg/mL) was recorded at ∼A_360_ nm wavelength. (B) XRD pattern of RL@ZnO nanoparticles for a typical wurtzite type polycrystals and particle size was found to be ∼27 nm. (C) SEM image of RL@ZnO nanoparticles demonstrates clearly the formation of aggregation of ZnO nanoparticles. (D) EDX image of RL@ZnO nanoparticles indicates the presence of zinc and oxygen molecules only.

As shown in Figure 4A, UV–vis spectrum of the RL@ZnO nanoparticles and CEMS077 RL. The spectrum of RL@ZnO nanoparticles (10 µg/mL) revealed a characteristic absorption peak at ∼A_360_ nm, which can be assigned to the intrinsic band-gap absorption of RL@ZnO nanoparticles. It was related to electron (e^−^) transitions from the valence band (Ev) to the conduction band (Ec) (O_2p_ → Zn_3d_) [26]. The peak also indicates the broad nano-sized particles distribution of RL@ZnO nanoparticles. While, RL shows the absorbance at A_230_ nm, it was due to the presence of the ring compounds in their composition. These results indicate that RL does not influence the absorption of RL@ZnO nanoparticles.

The crystal structure of RL@ZnO nanoparticles is depicted in the X-ray diffraction (XRD) spectrum with Cu Kα radiation (λ = 0.15418 nm). Obtained data revealed that the well resolved 11 XRD peaks were identified at 2θ = 31.11°, 34.23°, 35.61°, 47.13°, 56.15°, 62.18°, 65.65°, 67.59°, 69.21°, 72.18°, and 76.23° which are verified with the JCPDS card (No. 5-0664) for a typical wurtzite type polycrystals (Figure 4B). The presence of [100], [002], [102], [110], [103], [200], [112], [201], [004], and [202] planes indicate the absence of any impurity and suggest the high purity of RL@ZnO nanoparticles obtained by this method. The mean crystalline size of RL@ZnO nanoparticles was found to be ∼27 nm, determined by Debye–Scherrer formula. These results are in agreement with the previous report of Shoeb et al. [1]. Figure 4C shows the scanning electron microscopy (SEM) microphotographs and demonstrate clearly the formation of aggregation of RL@ZnO nanoparticles. Energy dispersive X-ray (EDX) analysis showed the elemental composition of RL@ZnO nanoparticles. EDX mapping results showed four peaks, which identify as zinc and oxygen and no other elemental impurities present in the synthesized nanomaterials (Figure 4D).

**Figure 5:**
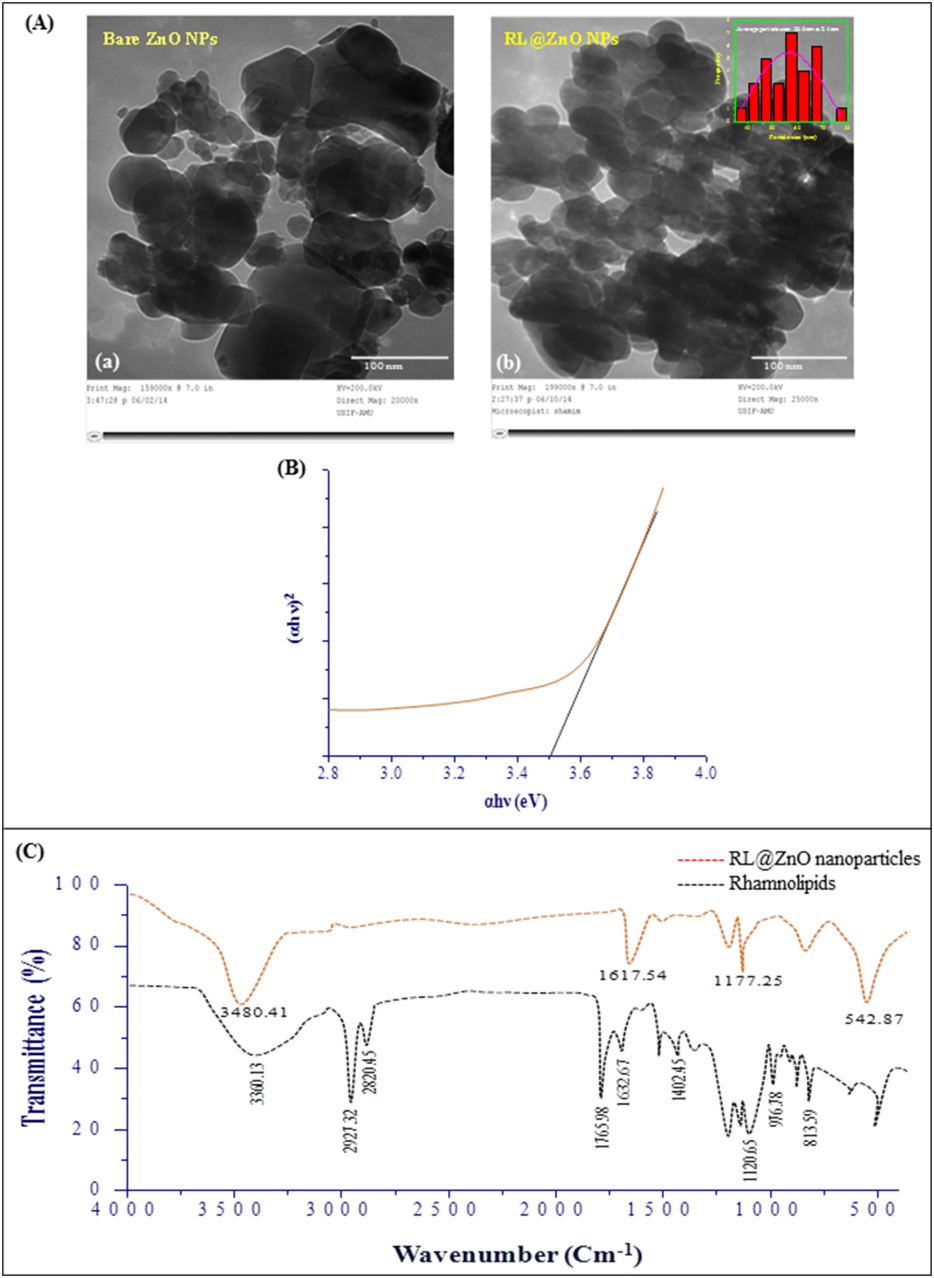
Characterization of RL@ZnO nanoparticles. (A) The representative TEM microphotographs of bare ZnO nanoparticles (a) and RL@ZnO nanoparticles (b) at an accelerating voltage of ∼200 kV. Inset of the figure (b) depicts the particle size analysis. (B) Band gap plot of RL@ZnO nanoparticles, calculated by Tauc plot formula. (C) FTIR spectra of RL extract (red line) and RL@ZnO nanoparticles (black line). The spectra shown are representatives of the three independent experiments.

The morphology and nanostructure of RL@ZnO nanoparticles was further characterized by transmission electron microscopy (TEM) analysis and results clearly showed that the RL@ZnO nanoparticles to be actually composed of several particles of different size grouped in clusters with bloody microphotograph (Figure 5A–b) as compared to bare nanoparticles (Figure 5A–a), it was due to encapsulation of nanoparticles by CEMS077 RLs. It can also be seen that these nanoparticles has a spherical structure with a particle size ranged from 35–80 nm. The mean particle size of RL@ZnO nanoparticles was calculated to be ∼57 nm using ImageJ processing and analysis software (http://imagej.nih.gov/ij/index.html) (Figure 5A inset). The TEM analysis also showed slight agglomeration of RL@ZnO nanoparticles due to Van der Waals forces between the nanoparticles.

**Figure 6:**
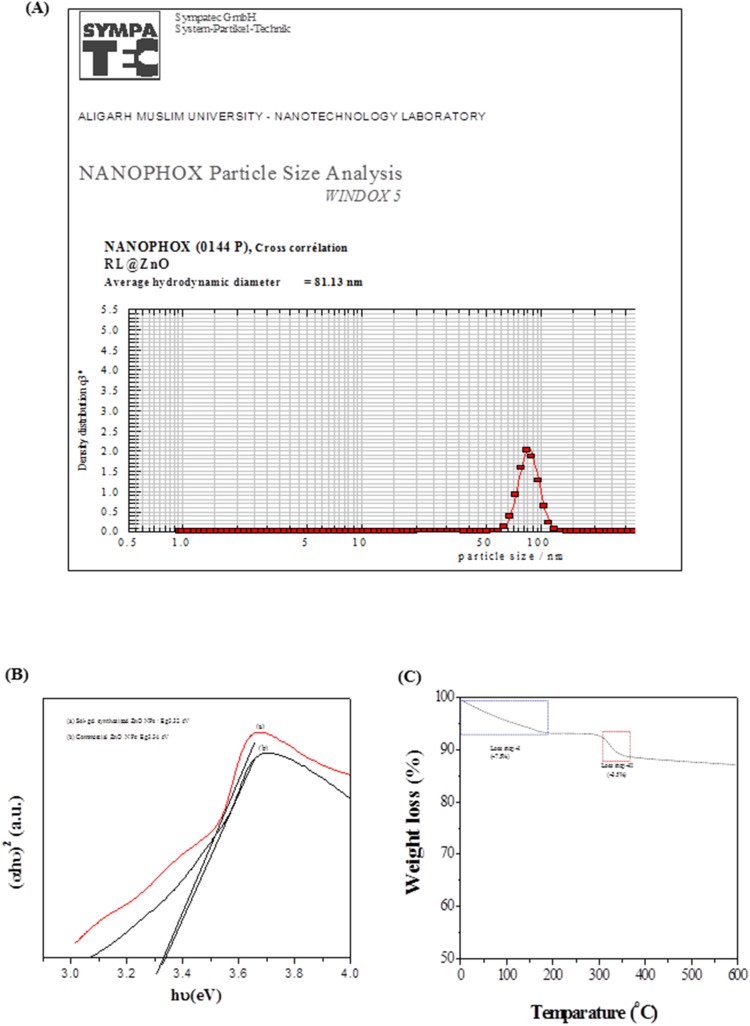
Determination of particle size and chemical properties of mfAgNPs. (A) Determination of particle size by DLS technique, (B) energy band gap of sol-gel and commercial ZnO nanoparticles upon the addition of RL, and (C) TGA curve.

The dynamic light scattering (DLS) measurement was performed for determination of the hydrodynamic diameters of RL@ZnO nanoparticles. The mean hydrodynamic diameter of RL@ZnO nanoparticles was found to be ∼81 nm (Figure 6A). The higher value of average size obtained in DLS measurement in comparison to the TEM analysis was observed possibly due to the (i) agglomeration of RL@ZnO nanoparticles and (ii) DLS measures the hydrodynamic radii of the nanoparticles, which includes the solvent layer at the interface.

The electronic structure of RL@ZnO nanoparticles was characterized on the basis of electronic band gap energy (Eg), which is essentially an energy interval between the Ev and the Ec, each of which has a high density of states [27]. Therefore, next we calculated the Eg of RL@ZnO nanoparticles (Figure 5B) by Tauc relationship as given below:
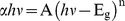

*α*, A, *h*, *v* and E_g_ are absorption coefficient (2.303A/t), absorbance, Planck’s constant, photon frequency, and electronic band gap, respectively. The value of n = 1/2, 3/2, 2 or 3 depending on the nature of electronic transition responsible for absorption and n = 1/2 for direct band gap semiconductor. The Eg of RL@ZnO nanoparticles was determined to be ∼3.5, which was the higher than bulk ZnO nanoparticles (E_g_ = 3.37 eV). The high value of Eg possibly attributed due to quantum confinement effect and surface modification of the nanoparticles by RLs. The Eg widening effect may be related to the influence of numerous factors including structural parameters (size and pH), carrier concentrations, and presence of defects (oxygen vacancies) that can lead to the Burstein Moss Shift [28]. The optical properties of ZnO are more interesting since the confinement of charge carriers in restricted volume of the small particles can lead to widening effects of Eg [1]. These results suggest that the CEMS077 RLs facilitate Eg widening effect by controlling nucleation and surface capping of nanoparticles [29]. The sugar and CH aliphatic moieties of RL are sufficient to form proper capping, resulting in the synthesis of smaller sized ZnO nanoparticles, so as to keep the wide Eg [30]. However, Eg of sol-gel synthesized and commercial ZnO nanoparticles upon the addition of same amount of RLs do not show the significant changes and determine to be ∼3.32 eV and 3.34 eV, respectively (Figure 6B).

Fourier transform infrared (FTIR) spectra revealed that the possible interactions between CEMS077 RLs and ZnO nanoparticles (Figure 5C). FTIR spectra of the RL showed important stretching vibration bands at 3360.13 cm^−1^, 2921.32 cm^−1^, 2820.45 cm^−1^, 1765.98 cm^−1^, 1632.67 cm^−1^, and 1400–800 cm^−1^, which are confirmed the glycolipid-type nature of the RLs [31]. The band at 3360.13 cm^−1^ denoted the presence of −OH stretching vibration and bands at 2921.32 cm^−1^ and 2820.45 cm^−1^ positions revealed the presence of −CH aliphatic stretching vibrations. The characteristic stretching vibration bands at 1765.98 cm^−1^ and 1632.67 cm^−1^ denoted the–C = O and –COO stretching vibrations of carbonyl group, respectively. The other bands at 1400 cm^−1^ and 800 cm^−1^ in the lower wavenumber absorption peaks confirmed the presence of ester carbonyl groups, which was corresponded to the presence of-C-O-deformation vibrations. FTIR spectrum also shows the characteristic vibration band at 542 cm^−1^, which was correspond to E_2_ mode of hexagonal ZnO wurtzite structure [1]. The vibration band at the position 542 cm^−1^ reflected to RL@ZnO nanoparticles stretching frequency of Zn-O bonds. The major vibration bands of RL in ZnO nanoparticles were shows the noticeable shifting and transmittance reduction, which is confirms the interaction between RL and ZnO nanoparticles. Previous reports also confirms that RLs can bind to metal nanoparticles by electrostatic attraction which occurs between free negatively charged carboxylate groups [24].

Thermal gravimetric analysis (TGA) of the RL@ZnO nanoparticles was performed and results are shown in Figure 6C. The presence of RLs on the surface of RL@ZnO nanoparticles was supported by TGA data which indicate two different mass loss behaviors. TGA curve revealed that the first weight loss (∼7.5%) at temperatures around <200°C was due to the loss of chemisorbed water. The second weight loss (∼3.5%) at ∼315°C is due to desorption and subsequent evaporation of RL from RL@ZnO nanoparticles.

### RLs increase the stability of ZnO nanoparticles

RLs have simple molecular structure, low molecular weight, and high affinity for the metals. Figure S2 in File S1 shows the stability of bare ZnO and RL@ZnO nanoparticles at room temperature over a period of 15 months, taking time of the synthesis studied here. Each time, we made 200 µg/mL aqueous solutions of both nanoparticles and recorded the change in UV–vis spectrum at A_360_ nm wavelength. The RL@ZnO nanoparticles showed no significant changes in absorbance and agglomeration up to 15 months of the storage. However, after 6 months the significant changes were recorded in bare ZnO nanoparticles. It is speculated that RLs are better capping ligands because of (i) the longer carbon chain and (ii) the fact that the double bond leads to a more disordered shell and thus facilitates dissolution. The RL with longer tails stabilizes nanoparticles better due to the fact that formation of the micelle-like aggregates on the surface of nanoparticles is easier for surfactant than the carboxymethyl cellulose [32]. Here, we propose that the CEMS077 RLs can be used for capping and stabilization of metal nanoparticles [24].

### RLs sustain the antioxidant activity of ZnO nanoparticles

**Figure 7:**
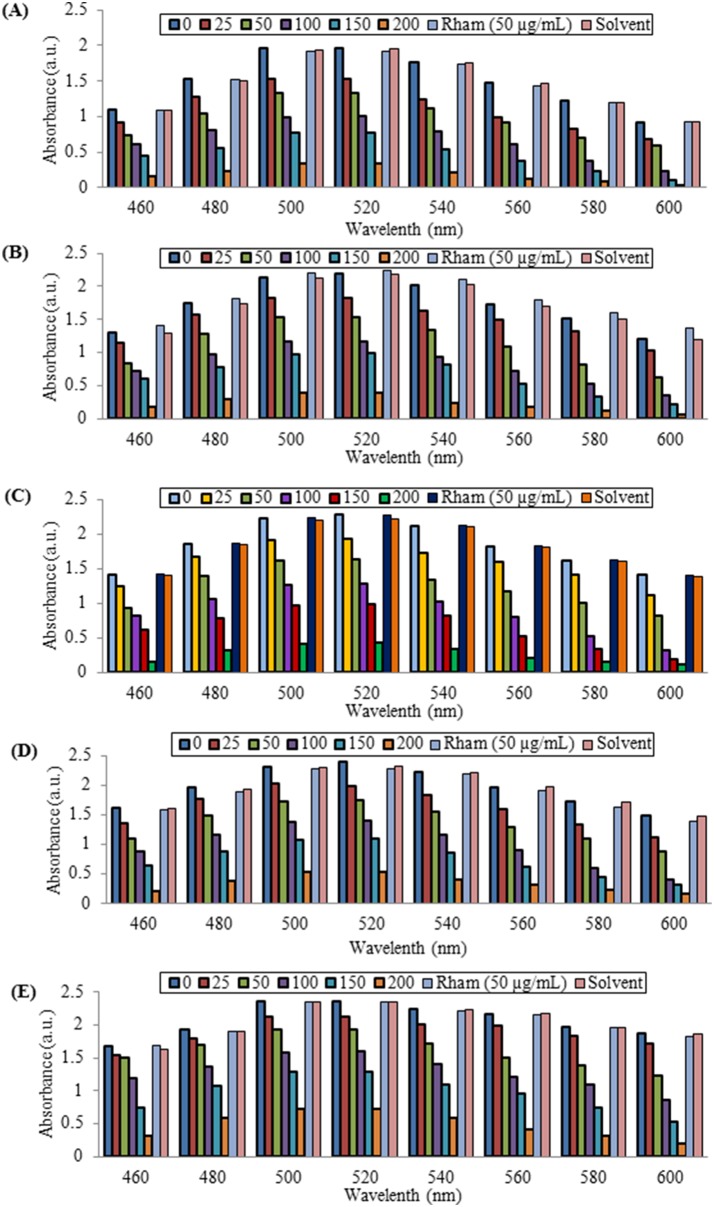
DPPH radical scavenging activity of RL@ZnO nanoparticles. RL@ZnO nanoparticles (0, 25, 50, 100, 150, and 200 mg/mL) were added to freshly prepared and stabilized DPPH solution (6×10^−5^ M), mixed and incubated at room temperature for 60 min. Then, DPPH radical scavenging potential was monitored by measuring the absorbance in the range A_460−600_ nm wavelength using a UV-vis spectrophotometer. The peak intensity at A_517_ nm gradually decreases in RL@ZnO nanoparticles. DPPH radical scavenging activity was measured at different time intervals: (A) 3, (B) 6, (C) 9, (D) 12, and (E) 15 months of the storage of RL@ZnO nanoparticles.

Next, we determined whether RLs isolated from CEMS077 could retain the functional attribute of ZnO nanoparticles with special reference to their antioxidant activity. For this, we used DPPH free radical solution and first determined its stability at room temperature up to 2.5 h. There is no change of color and the absorption intensity was recorded at A_517_ nm. DPPH is a stable nitrogen-centered, lipophilic free radical that is widely used for evaluating the antiradical activity of antioxidants in a relatively short time as compared to the other methods [33]. The color of DPPH solution gradually changed from deep violet to pale yellow in the presence of RL@ZnO nanoparticles by transferring electron density located at oxygen to the odd electron located at nitrogen atom in DPPH. The peak intensity at A_517_ nm gradually decreased in RL@ZnO nanoparticles and no significant changes in the antioxidant activity were recorded up to 15 months (Figure 7A–E). The decrease of peak intensity gives the evidence toward the antiradical property of RL@ZnO nanoparticles. We have observed the decline antiradical capacity by 6.9% of 200 µg/mL RL@ZnO nanoparticles up to15 months of storage as compared to 0 month; whereas it was declined by 87.8% in bare ZnO nanoparticles at the same concentration (Figure S3A–E in File S1).

**Figure 8:**
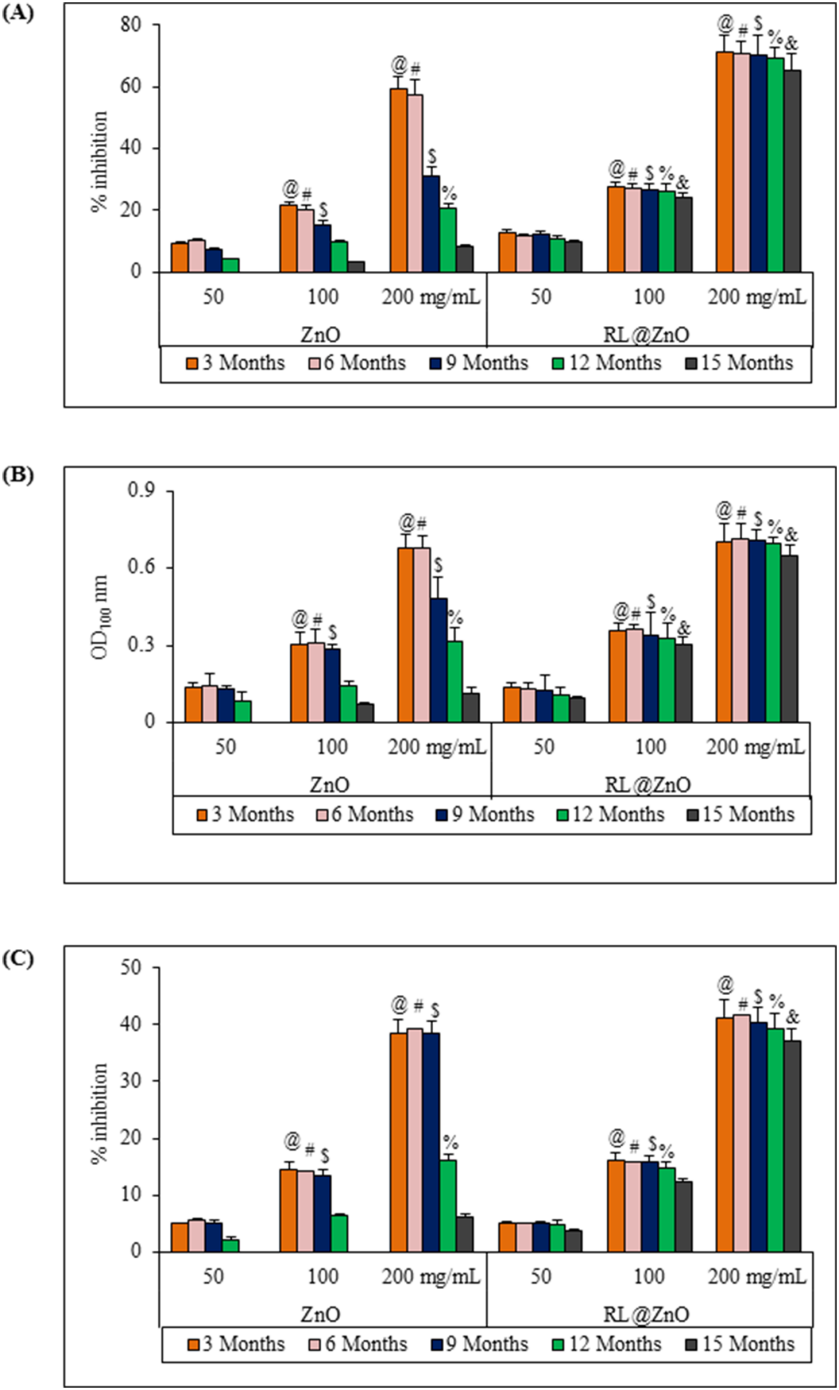
Antioxidant potential of RL@ZnO nanoparticles. (A) To determine the antioxidant activity of bare ZnO and RL@ZnO nanoparticles, autoxidation of β-carotene and linoleic acid coupled reaction mixture was used. Inhibition of β-carotene oxidation in the presence of different concentrations of these nanoparticles was monitored at A_470_ nm spectrophotometrically. Control contained distilled water only in place of nanoparticles. (B) For measurement of reducing potential, phosphate buffer (2.5 mL; 0.1 M; pH 6.6) and 1% potassium ferricyanide (2.5 mL) were mixed with the indicated concentrations of bare ZnO and RL@ZnO nanoparticles and incubated at 50°C for 20 min. After addition of 0.1% FeCl_3_ (0.5 mL), the absorbance was recorded at A_700_ nm. (C) ^•^OH radical scavenging activity of bare ZnO and RL@ZnO nanoparticles was determined by using deoxyribose assay. Fenton’s reaction (50 µM ascorbic acid, 20 µM FeCl_3_, 2 mM EDTA, 1.42 mM H_2_O_2_), 2.8 mM deoxyribose sugar and indicated concentration of test nanoparticles were mixed and incubated at 37°C for 1 h. After addition of 1% TBA, mixture was heated at 90°C for 15 min and then, absorbance was recorded at A_532_ nm. Data represent mean ± SD. Different letters: @, #, $, %, and & significantly different from their respective controls according to *post hoc* comparison (LSD-test) (*P*<0.01).

Next, auto-oxidation of β-carotene and linoleic acid coupled reaction method was used to assess the antioxidant activity of RL@ZnO and bare ZnO nanoparticles. Antioxidant activities decreased by 5.8% and 49% in RL@ZnO and bare ZnO nanoparticles, respectively (Figure 8A). Here, we assume that the observed long term stability of RL@ZnO nanoparticles might be due to encapsulation of nanoparticles by CEMS077 RLs which prevent erosion and oxidation of core components (i.e. Zn^+^ ions). These assumptions are further supported by the fact that RL@ZnO nanoparticles exhibited long term reducing potential than the bare ZnO nanoparticles. We determined reduction capability based on the reduction of Fe^3+^ → Fe^2+^in the presence of nanoparticles and monitored at A_700_ nm wavelength. Similarly, non-significant decline was examined by 9.1%, when used 200 µg/mL of RL@ZnO nanoparticles, but a huge decline was recorded by 83% in bare ZnO nanoparticles (Figure 8B). The strong decrement in the reducing potential of bare ZnO nanoparticles further confirms the role of RLs in stabilization of RL@ZnO nanoparticles.

Although, RL@ZnO nanoparticles exhibited long term antioxidant activity than bare ZnO nanoparticles, however this potential of RL@ZnO nanoparticles is it still needed to confirm against most reactive free radical species including ^•^OH and O_2_^•−^. The ^•^OH radical is a most damaging ROS in chemistry due to its high reactive potential [34]. It can abstract hydrogen from thiols, leading the formation of sulphur radicals capable to combine with oxygen to generate oxysulfur radicals [33]. In this study, the ^•^OH radical scavenging activity was measured in order to clarify whether our synthesized RL@ZnO nanoparticles inhibit the damage of deoxyribose sugar by scavenging ^•^OH radicals. Figure 8C shows no significant decline in deoxyribose sugar protective potential of RL@ZnO nanoparticles over a period of 15 months. As evident from the data, 200 µg/mL of bare ZnO nanoparticles lost by 86.4% ^•^OH radical scavenging activity, when compared to 0 months, while in RL@ZnO nanoparticles, it was only 4.0%. Similarly, we also observed non-significant decline in lipid peroxidation protective potential by 10.8% and O_2_^•−^ radical scavenging activity by 7.7% in RL@ZnO nanoparticles, while these decrements were observed more in bare ZnO nanoparticles by 46.1 and 51.1%, respectively (Figure S4A and B in File S1). All decline effects in bare ZnO nanoparticles were observed after 9 months of the storage in comparison with 0 months. The antioxidant activity of ZnO nanoparticles may be due to the transfer of electron density located at oxygen to the odd electron located at outer orbits of oxygen in ^•^OH and O_2_^•−^ radicals. Retention of antioxidant activity of RL@ZnO nanoparticles may be due to the surface protection by RL, resulting in stabilization of nanoparticles. Our study establishes the possible role of *P*. *aeruginosa* RLs for encapsulation and stabilization of ZnO nanoparticles.

## Conclusions

In the present study, RL producing *P*. *aeruginosa* CEMS077 has been used for the first time to synthesize stable antioxidant ZnO nanoparticles. Antioxidant and free radical scavenging activities elucidate that ZnO nanoparticles can be used as a promising antioxidant in biological system. Moreover, CEMS077 RLs have significance in nanotechnology for the large-scale production of surface protective ZnO nanoparticles, exploiting the low-cost environmental friendly approach for synthesis of stabilized nanomaterials.

## Experimental Procedures

### Materials

Zinc nitrite (ZnNO_3_) and sodium hydroxide pellets were procured from SRL, India. DPPH, 2-deoxyribose sugar, thiobarbituric acid (TBA), riboflavin, and nitro blue tetrazolium (NBT) were purchased from Sigma–Aldrich. Ascorbic acid, β-carotene, tween-40, and linoleic acid were obtained from MP Biomedicals. All the reagents and solvents used were of analytical grade.

### Isolation of RL producing bacterial strain

RL producing bacterial strain CEMS077 was isolated from the roots of Congress Grass (*Parthenium hysterophorus*), Aligarh, Uttar Pradesh, India (Latitude of 27° 30′; Longitude of 79° 40′). No specific permission was required for this location. The stock culture of CEMS077 was maintained on nutrient agar (NA; HiMedia, India) slant containing 1% glycerol. The working culture of CEMS077 was prepared by streaking NA plates from its stock culture and plate was incubated at 30±1°C for 18–24 h.

### Production and isolation of RLs

To promote RL production, the nutrient broth (NB; HiMedia, India) was modified with 3% glycerol to prepare glycerol supplemented NB (GSNB; HiMedia) medium. The pH of the GSNB medium was adjusted to 7.0±0.2 and then sterilized at 15 lbs pressure for 15 min. The sterilized GSNB medium was inoculated with 0.5% overnight culture of CEMS077 and incubated under static condition at 30±1°C for 96 h. To isolate RLs, CEMS077 culture was centrifuged at 12,000 rpm for 15 min. The obtained supernatant was collected in the sterile separating funnel, acidified by 12N HCl (pH 2.0) to precipitate RLs, and incubated at 4°C overnight. The brown color precipitate RLs were recovered by centrifugation at 10,000 rpm for 10 min and extracted with equal volume of chloroform-ethanol (2∶1) mixture. The solvents were then evaporated and extracted RLs showed an oil-like appearance. The extracted RLs were dissolved in an appropriate volume of methanol and transferred to a previously weighed beaker. Methanol was evaporated and stored at 4°C until further use.

### 16S rRNA gene sequencing

The strain CEMS077 was grown in NB broth at 30±1°C. The cells were harvested after 24 h of incubation and processed immediately for isolation of DNA using the CTAB method. The gene coding for 16S rRNA was amplified by employing specific primers. Sequencing and data analysis was done according to the method reported recently by Singh et al. [9].

### Biosynthesis of RL@ZnO nanoparticles

RL@ZnO nanoparticles were synthesized and stabilized by following the method of Singh et al. [9]. Typically, 50 mL of RL (50 mg/L) at 1.1-fold critical micelle concentration was mixed with 50 mL of 1 mM ZnNO_3._ This solution was incubated at 80°C for 30 min under vigorous stirring to form RL–Zn complex solution. To obtain the colloidal RL@ZnO nanoparticles, few drops of 1 M NaOH solution were added under vigorous stirring. The color change was monitored by visual inspection and by measuring absorbance by UV–Vis spectrophotometer (PerkinElmer Life and Analytical Sciences, CT, USA). The resulting white precipitate was obtained by centrifugation at 5000 rpm for 10 min, washed with sterilized RO water and dried at 70°C under vacuum oven. The dried RL@ZnO nanoparticles were grounded and stored in amber colour container until further use.

### Characterization of RL@ZnO nanoparticles

The synthesis of RL@ZnO nanoparticles was monitored by measuring UV–vis absorption spectra (Perkin Elmer Life and Analytical Sciences, CT, USA) in the range 200 to 800 nm wavelength [6]. Crystalline metallic ZnO nanoparticles were examined using a Mini Flex TM II bench top XRD system (Rigaku Corporation, Tokyo, Japan) equipped with Cu Kα radiation (λ = 1.54 Å) source using Ni as filter at a setting of 30 kV/30 mA. The diffracted intensities were recorded from 20° to 80° at 2θ angles [1]. The size and shape of RL@ZnO nanoparticles was determined using a JEOL- JSM-6510LV SEM machine (Tokyo, Japan) and operated at an accelerating voltage of 20 kV. The elemental analysis was carried out on the Oxford Instrument INCA x-sight EDX spectrometer equipped with SEM. TEM analysis of the RL@ZnO nanoparticles was done using a JEOL, Tokyo TEM instrument with an accelerating voltage of 200 kV [1]. FTIR spectra of RL extract powder and ZnO nanoparticles was obtained in the range 4,000 to 400 cm^−1^ with a PerkinElmer FTIR spectrophotometer, by potassium bromide (KBr) pellet method [9]. Spectroscopic grade KBr was used in the ratio of 1∶100 and spectrum was recorded in the diffuse reflectance mode at a resolution of 4 cm^−1^ in KBr pellets. Thermal analysis of the RL@ZnO nanoparticles powder was carried out on a PerkinElmer TGA machine.

### Antioxidant activity assays

Antioxidant activity (AOA) of the RL@ZnO nanoparticles was examined by autoxidation of β-carotene and linoleic acid coupled reaction method as described elsewhere [33]. DPPH free radical was used to determine the free radical scavenging activity [33]. Reducing capacity of the RL@ZnO nanoparticles was assessed by ferric reducing antioxidant power assay and results were expressed as concentration *vs* optical density at A_700_ nm wavelength [35]. The deoxyribose assay was used to determine the ^•^OH radical scavenging property of the test material [36]. A thiobarbituric acid-reactive substances (TBARS) assay was used to measure the inhibitory degree of RL@ZnO nanoparticles on lipid peroxidation using egg yolk homogenate as a lipid rich template [37]. The O_2_^•−^ radical scavenging activity was examined based on the capacity of the antioxidant to inhibit the photochemical reduction of NBT [38].

### Ethics statement

The location that we collected rhizospheric soil samples was declared as an uncultivated land, so it did not need to get the specific permission. The host plant *P. hysterophorus* did not belong to the category of endangered or protected plant, because it is a weed. Moreover, we used sampling procedure that did not harm the plant diversity of the location.

### Statistical analysis

Results presented in tables and graphs were reported as means ± standard deviation (SD) of at least three replicates of the same concentration. Data were subjected to one-way analysis of variance (ANOVA) and the least significant difference (LSD) between the extracts at *P<*0.01 was calculated by *post-hoc* comparison test (SPSS11.5).

## Supporting Information

File S1 **Figure S1–S4.**
(PPTX)

## References

[1] Shoeb M, Braj RS, Javed AK, Wasi K, Brahma NS, et al (2013) ROS-dependent anticandidal activity of zinc oxide nanoparticles synthesized by using egg albumen as a biotemplate. Adv Nat Sci Nanosci Nanotechnol 4: 035015.

[2] Nel A, Xia T, Madler L, Li N (2006) Toxic potential of materials at the nanolevel. Science 311: 622–627. 16456071 10.1126/science.1114397

[3] Bowen WR, Lovitt R, Wright C (2000) Application of atomic force microscopy to the study of micromechanical properties of biological materials. Biotechnol Lett 22: 893–903.

[4] Avouris P (1995) Manipulation of matter at the atomic and molecular levels. Acc Chem Res 28: 95–102.

[5] Ansari SA, Husain Q, Qayyum S, Azam A (2011) Designing and surface modification of zinc oxide nanoparticles for biomedical applications. Food Chem Toxicol 49: 2107–2115. 21645581 10.1016/j.fct.2011.05.025

[6] Singh BR, Dwivedi S, Al-Khedhairy AA, Musarrat J (2011) Synthesis of stable cadmium sulfide nanoparticles using surfactin produced by *Bacillus amyloliquifaciens* strain KSU-109. Colloids Surf B Biointerfaces 85: 207–213. 21435848 10.1016/j.colsurfb.2011.02.030

[7] Kharissova OV, Dias HV, Kharisov BI, Perez BO, Perez VM (2013) The greener synthesis of nanoparticles. Trends Biotechnol 31: 240–248. 23434153 10.1016/j.tibtech.2013.01.003

[8] Bansal V, Poddar P, Ahmad A, Sastry M (2006) Room-temperature biosynthesis of ferroelectric barium titanate nanoparticles. J Am Chem Soc 128: 11958–11963. 16953637 10.1021/ja063011m

[9] Singh BR, Singh BN, Khan W, Singh HB, Naqvi AH (2012) ROS-mediated apoptotic cell death in prostate cancer LNCaP cells induced by biosurfactant stabilized CdS quantum dots. Biomaterials 33: 5753–5767. 22594971 10.1016/j.biomaterials.2012.04.045

[10] Bansal V, Rautaray D, Bharde A, Ahire K, Sanyal A, et al (2005) Fungus-mediated biosynthesis of silica and titania particles. J Mat Chem 15: 2583–2589.

[11] Banat IM, Franzetti A, Gandolfi I, Bestetti G, Martinotti MG, et al (2010) Microbial biosurfactants production, applications and future potential. Appl Microbiol Biotechnol 87: 427–444. 20424836 10.1007/s00253-010-2589-0

[12] Li J, Guo D, Wang X, Wang H, Jiang H, et al (2010) The photodynamic effect of different size ZnO nanoparticles on cancer cell proliferation in vitro. Nanoscale Res Lett 5: 1063–1071. 20671778 10.1007/s11671-010-9603-4 PMC2893699

[13] Premanathan M, Karthikeyan K, Jeyasubramanian K, Manivannan G (2011) Selective toxicity of ZnO nanoparticles toward Gram-positive bacteria and cancer cells by apoptosis through lipid peroxidation. Nanomedicine 7: 184–192. 21034861 10.1016/j.nano.2010.10.001

[14] Das D, Nath BC, Phukon P, Kalita A, Dolui SK (2013) Synthesis of ZnO nanoparticles and evaluation of antioxidant and cytotoxic activity. Colloids Surf B Biointerfaces 111C: 556–560. 10.1016/j.colsurfb.2013.06.041 23891844

[15] Prakash D, Suri S, Upadhyay G, Singh BN (2007) Total phenol, antioxidant and free radical scavenging activities of some medicinal plants. Int J Food Sci Nutr 58: 18–28. 17415953 10.1080/09637480601093269

[16] Singh BN, Singh BR, Singh RL, Prakash D, Dhakarey R, et al (2009) Oxidative DNA damage protective activity, antioxidant and anti-quorum sensing potentials of *Moringa oleifera* . Food Chem Toxicol 47: 1109–1116. 19425184 10.1016/j.fct.2009.01.034

[17] Singh BN, Singh BR, Sarma BK, Singh HB (2009) Potential chemoprevention of *N*-nitrosodiethylamine-induced hepatocarcinogenesis by polyphenolics from *Acacia nilotica* bark. Chem Biol Interact 181: 20–28. 19446540 10.1016/j.cbi.2009.05.007

[18] Prakash D, Upadhyay G, Singh BN, Singh HB (2007) Antioxidant and free radical-scavenging activities of seeds and agri-wastes of some varieties of soybean (*Glycine max*). Food Chem 104: 783–790.

[19] Singh BN, Singh BR, Singh RL, Prakash D, Sarma BK, et al (2009) Antioxidant and anti-quorum sensing activities of green pod of *Acacia nilotica* L. Food Chem Toxicol. 47: 778–786. 10.1016/j.fct.2009.01.009 19168114

[20] Singh BN, Singh BR, Singh RL, Prakash D, Singh DP, et al (2009) Polyphenolics from various extracts/fractions of red onion (*Allium cepa*) peel with potent antioxidant and antimutagenic activities. Food Chem Toxicol 47: 1161–1167. 19425188 10.1016/j.fct.2009.02.004

[21] Bansal V, Rautaray D, Ahmad A, Sastry M (2004) Biosynthesis of zirconia nanoparticles using the fungus *Fusarium oxysporum* . J Mat Chem 14: 3303–3305.

[22] Sanyal A, Rautaray D, Bansal V, Ahmad A, Sastry M (2005) Heavy-metal remediation by a fungus as a means of production of lead and cadmium carbonate crystals. Langmuir 21: 7220–7224. 16042445 10.1021/la047132g

[23] Bansal V, Kumar M, Dalela M, Brahmne HG, Singh H (2014) Evaluation of synergistic effect of biodegradable polymeric nanoparticles and aluminum based adjuvant for improving vaccine efficacy. Int J Pharm 471: 377–384. 24939616 10.1016/j.ijpharm.2014.05.061

[24] Hazra C, Kundu D, Chaudhari A, Jana T (2013) Biogenic synthesis, characterization, toxicity and photocatalysis of zinc sulfide nanoparticles using rhamnolipids from *Pseudomonas aeruginosa* BS01 as capping and stabilizing agent. J Chem Technol Biotechnol 88: 1039–1048.

[25] Sangeetha J, Thomas S, Arutchelvi J, Doble M, Philip J (2013) Functionalization of iron oxide nanoparticles with biosurfactants and biocompatibility studies. J Biomed Nanotechnol 9: 751–764. 23802405 10.1166/jbn.2013.1590

[26] Zak AK, Abrishami ME, Majid WHA, Yousefi R, Hosseini SM (2011) Effects of annealing temperature on some structural and optical properties of ZnO nanoparticles prepared by a modified sol–gel combustion method. Ceram Int 37: 393–398.

[27] Zhang H, Ji Z, Xia T, Meng H, Low-Kam C, et al (2012) Use of metal oxide nanoparticle band gap to develop a predictive paradigm for oxidative stress and acute pulmonary inflammation. ACS Nano 6: 4349–4368. 22502734 10.1021/nn3010087 PMC4139054

[28] Ahmed AS, Shafeeq MM, Singla ML, Tabassum S, Naqvi AH, et al (2011) Band gap narrowing and fluorescence properties of nickel doped SnO_2_ nanoparticles. J Lumin 131: 1–6.

[29] Singla ML, Shafeeq MM, Kumar M (2009) Optical characterization of ZnO nanoparticles capped with various surfactants. J Lumin 129: 434–438.

[30] Kathiravan A, Paramaguru G, Renganathan R (2009) Study on the binding of colloidal zinc oxide nanoparticles with bovine serum albumin. J Mol Struc 934: 129–137.

[31] Saikia JP, Bharali P, Konwar BK (2013) Possible protection of silver nanoparticles against salt by using rhamnolipid. Colloids Surf B Biointerfaces 104: 330–332. 23290768 10.1016/j.colsurfb.2012.10.069

[32] Mehta SK, Kumar S, Chaudhary S, Bhasin KK, Gradzielski M (2009) Evolution of ZnS nanoparticles via facile CTAB aqueous micellar solution route: A study on controlling parameters. Nanoscale Res Lett 4: 17–28. 20592958 10.1007/s11671-008-9196-3 PMC2893752

[33] Singh HB, Singh BN, Singh SP, Nautiyal CS (2010) Solid-state cultivation of *Trichoderma harzianum* NBRI-1055 for modulating natural antioxidants in soybean seed matrix. Bioresour Technol 101: 6444–6453. 20363120 10.1016/j.biortech.2010.03.057

[34] Halliwell B, Murcia MA, Chirico S, Aruoma OI (1995) Free radicals and antioxidants in food and in vivo: what they do and how they work. Crit Rev Food Sci Nutr 35: 7–20. 7748482 10.1080/10408399509527682

[35] Apáti P, Szentmihályi K, Kristó ST, Papp I, Vinkler P, et al (2003) Herbal remedies of *Solidago*–correlation of phytochemical characteristics and antioxidative properties. J Pharm Biomed Anal 32: 1045–1053. 12899992 10.1016/s0731-7085(03)00207-3

[36] Halliwell B, Gutteridge JM, Aruoma OI (1987) The deoxyribose method: a simple “test-tube” assay for determination of rate constants for reactions of hydroxyl radicals. Anal Biochem 165: 215–219. 3120621 10.1016/0003-2697(87)90222-3

[37] Ohkawa H, Ohishi N, Yagi K (1979) Assay for lipid peroxides in animal tissues by thiobarbituric acid reaction. Anal Biochem 95: 351–358. 36810 10.1016/0003-2697(79)90738-3

[38] Beauchamp C, Fridovich I (1971) Superoxide dismutase: improved assays and an assay applicable to acrylamide gels. Anal Biochem 44: 276–287. 4943714 10.1016/0003-2697(71)90370-8

