# Supplementary material for: Biosynthesis of Stable Antioxidant ZnO Nanoparticles by Pseudomonas aeruginosa Rhamnolipids
Source: PLoS One. 2014 Sep 4;9(9):e106937. doi: 10.1371/journal.pone.0106937 (PMC4154833; doi:10.1371/journal.pone.0106937)
Supplement: File S1 — Figure S1–S4. (PPTX) [file pone.0106937.s001.pptx]

## Slide 1
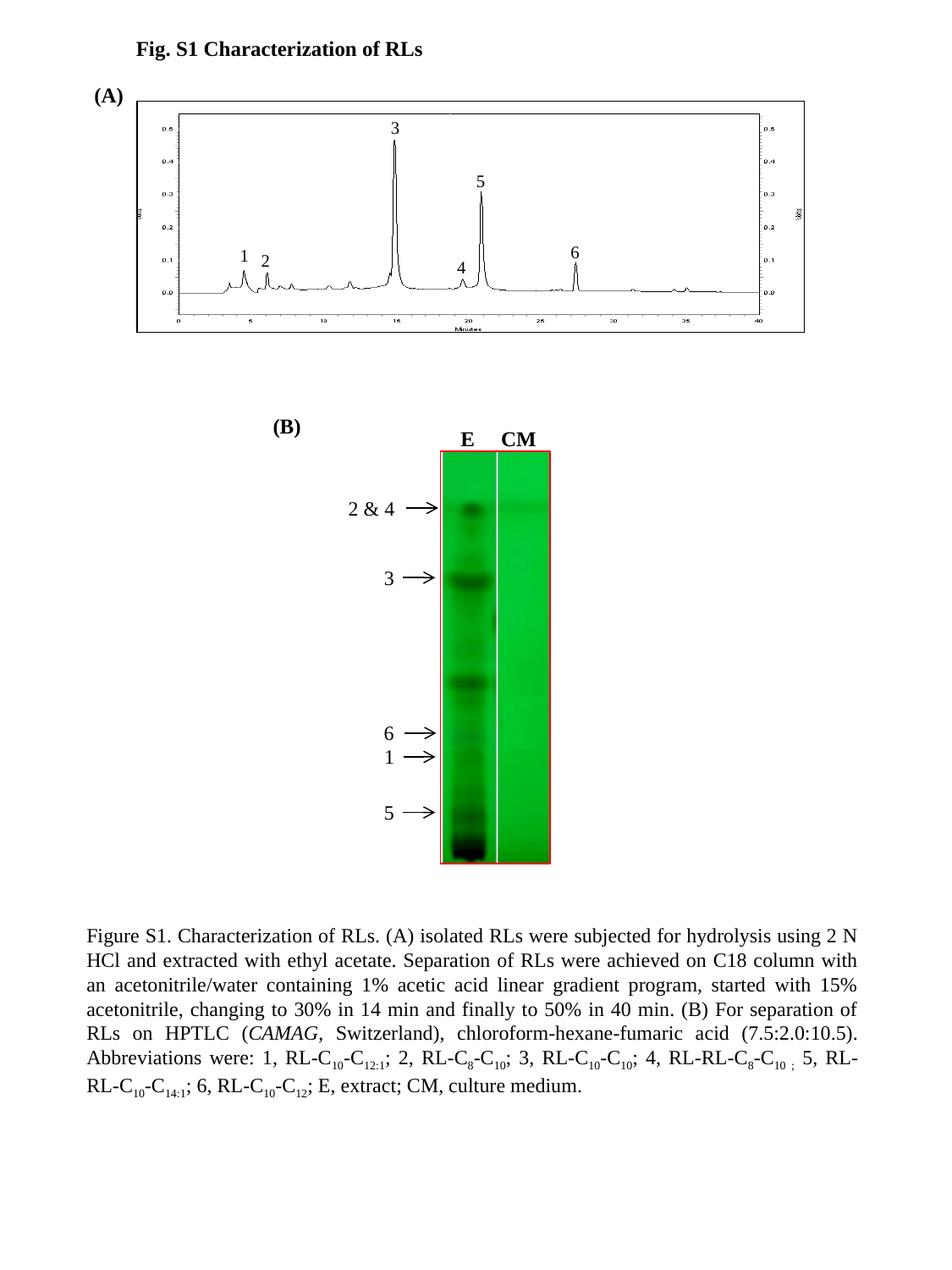

Fig. S1 Characterization of RLs
(A)
3
5
6
1
2
4
(B)
 E CM
2 & 4
3
6
1
5
Figure S1. Characterization of RLs. (A) isolated RLs were subjected for hydrolysis using 2 N HCl and extracted with ethyl acetate. Separation of RLs were achieved on C18 column with an acetonitrile/water containing 1% acetic acid linear gradient program, started with 15% acetonitrile, changing to 30% in 14 min and finally to 50% in 40 min. (B) For separation of RLs on HPTLC (CAMAG, Switzerland), chloroform-hexane-fumaric acid (7.5:2.0:10.5). Abbreviations were: 1, RL-C10-C12:1; 2, RL-C8-C10; 3, RL-C10-C10; 4, RL-RL-C8-C10 ; 5, RL-RL-C10-C14:1; 6, RL-C10-C12; E, extract; CM, culture medium.

## Slide 2
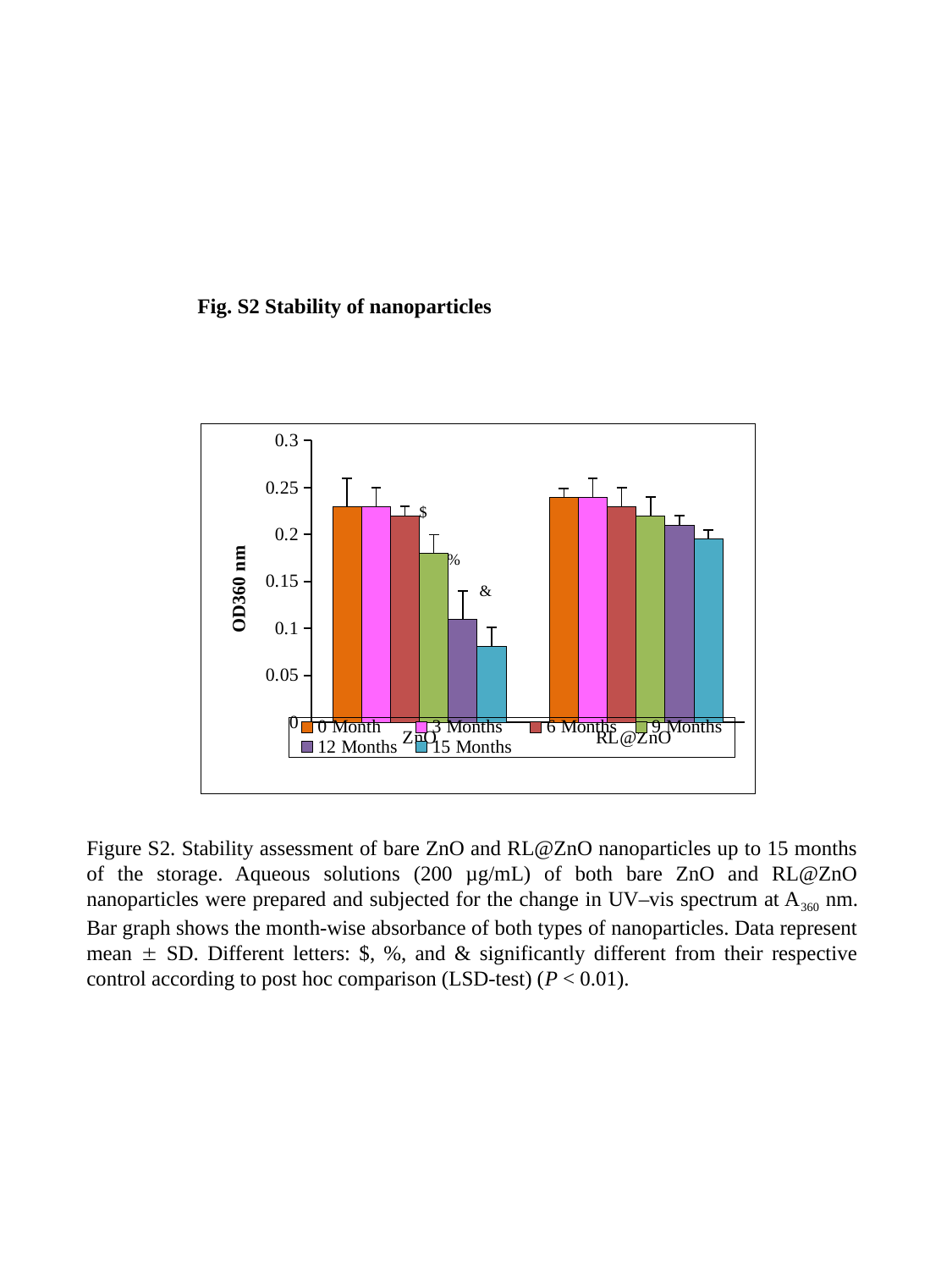

Fig. S2 Stability of nanoparticles
### Chart
| Category | 0 Month | 3 Months | 6 Months | 9 Months | 12 Months | 15 Months |
|---|---|---|---|---|---|---|
| ZnO | 0.23 | 0.23 | 0.22000000000000003 | 0.18000000000000008 | 0.11000000000000001 | 0.08100000000000002 |
| RL@ZnO | 0.24000000000000007 | 0.24000000000000007 | 0.23 | 0.22000000000000003 | 0.21000000000000008 | 0.19500000000000003 |$
%
&
Figure S2. Stability assessment of bare ZnO and RL@ZnO nanoparticles up to 15 months of the storage. Aqueous solutions (200 µg/mL) of both bare ZnO and RL@ZnO nanoparticles were prepared and subjected for the change in UV–vis spectrum at A360 nm. Bar graph shows the month-wise absorbance of both types of nanoparticles. Data represent mean ± SD. Different letters: $, %, and & significantly different from their respective control according to post hoc comparison (LSD-test) (P < 0.01).

## Slide 3
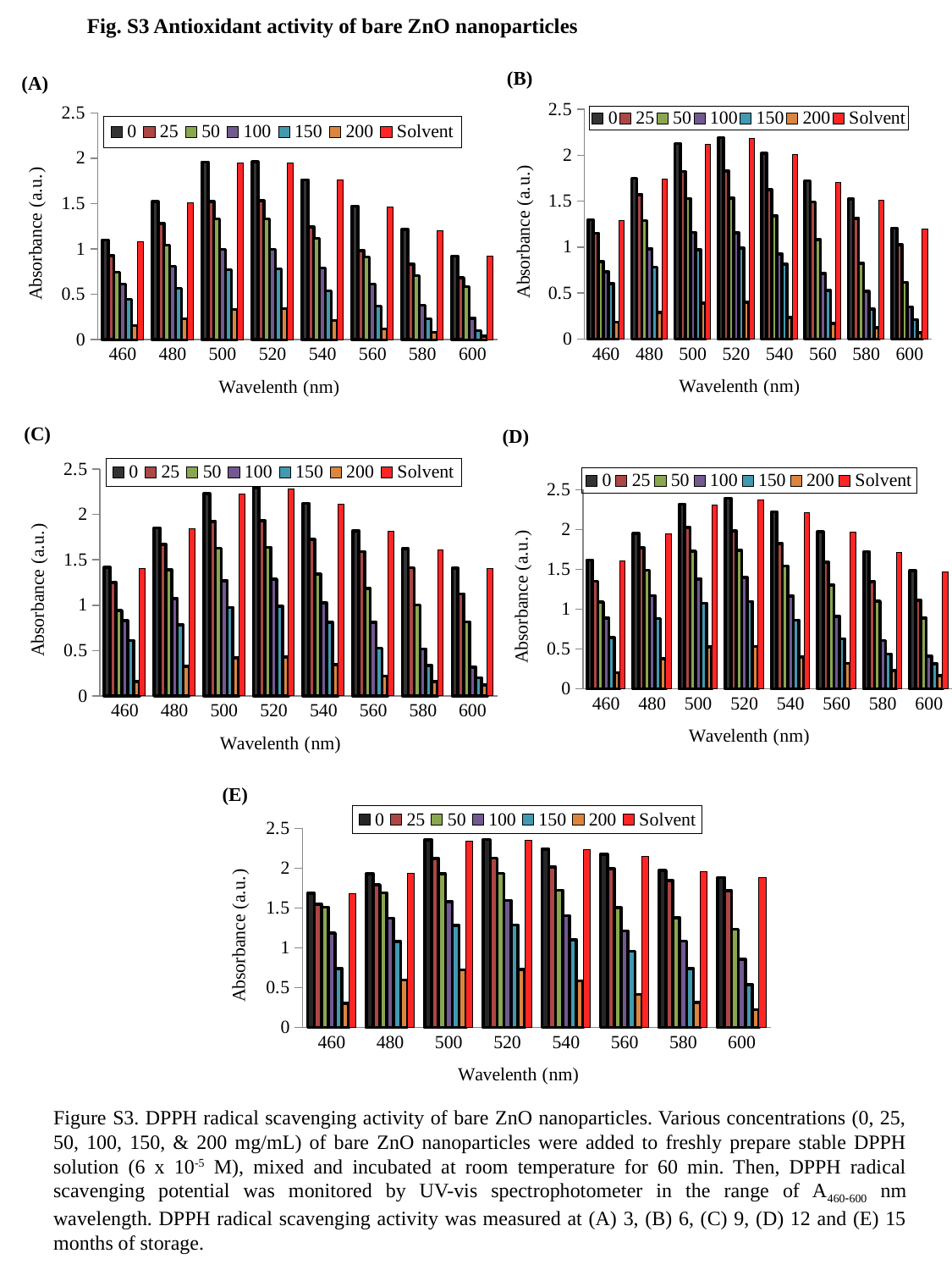

Fig. S3 Antioxidant activity of bare ZnO nanoparticles
### Chart
| Category | 0 | 25 | 50 | 100 | 150 | 200 | Solvent |
|---|---|---|---|---|---|---|---|
| 460 | 1.2969999999999993 | 1.151 | 0.8410000000000003 | 0.7280000000000003 | 0.6020000000000003 | 0.18100000000000008 | 1.292 |
| 480 | 1.7509999999999992 | 1.572 | 1.29 | 0.981 | 0.782 | 0.28900000000000015 | 1.744 |
| 500 | 2.127 | 1.823 | 1.528 | 1.161 | 0.9730000000000003 | 0.39100000000000024 | 2.12 |
| 520 | 2.1919999999999997 | 1.831 | 1.536 | 1.157 | 0.987 | 0.39900000000000024 | 2.184 |
| 540 | 2.022 | 1.623 | 1.342 | 0.925 | 0.813 | 0.232 | 2.0119999999999987 |
| 560 | 1.722 | 1.492 | 1.082 | 0.7120000000000003 | 0.528 | 0.168 | 1.7029999999999994 |
| 580 | 1.5229999999999992 | 1.3129999999999993 | 0.821 | 0.517 | 0.3280000000000002 | 0.12100000000000002 | 1.5129999999999992 |
| 600 | 1.206 | 1.0249999999999992 | 0.6160000000000003 | 0.34900000000000014 | 0.21100000000000008 | 0.06900000000000003 | 1.2 |
### Chart
| Category | 0 | 25 | 50 | 100 | 150 | 200 | Solvent |
|---|---|---|---|---|---|---|---|
| 460 | 1.097 | 0.924 | 0.7430000000000003 | 0.6120000000000003 | 0.443 | 0.15400000000000008 | 1.081 |
| 480 | 1.5209999999999992 | 1.282 | 1.042 | 0.805 | 0.563 | 0.234 | 1.5129999999999992 |
| 500 | 1.957 | 1.524 | 1.328 | 0.993 | 0.7740000000000004 | 0.33200000000000024 | 1.9460000000000006 |
| 520 | 1.959 | 1.53 | 1.333 | 0.999 | 0.7780000000000004 | 0.33900000000000025 | 1.947 |
| 540 | 1.762 | 1.242 | 1.119 | 0.788 | 0.542 | 0.21400000000000008 | 1.7569999999999992 |
| 560 | 1.472 | 0.983 | 0.913 | 0.6120000000000003 | 0.37300000000000016 | 0.11600000000000002 | 1.462 |
| 580 | 1.214 | 0.8340000000000003 | 0.7020000000000003 | 0.3820000000000002 | 0.227 | 0.085 | 1.2049999999999994 |
| 600 | 0.921 | 0.683 | 0.583 | 0.23500000000000001 | 0.09900000000000005 | 0.039000000000000014 | 0.917 |(B)
(A)
### Chart
| Category | 0 | 25 | 50 | 100 | 150 | 200 | Solvent |
|---|---|---|---|---|---|---|---|
| 460 | 1.4169999999999994 | 1.2509999999999992 | 0.941 | 0.8280000000000003 | 0.6120000000000003 | 0.161 | 1.4 |
| 480 | 1.851 | 1.6719999999999993 | 1.3900000000000001 | 1.071 | 0.782 | 0.32900000000000024 | 1.838 |
| 500 | 2.227 | 1.923 | 1.6279999999999992 | 1.2709999999999992 | 0.9730000000000003 | 0.4210000000000002 | 2.221 |
| 520 | 2.292 | 1.931 | 1.6359999999999992 | 1.2869999999999993 | 0.987 | 0.4270000000000002 | 2.285 |
| 540 | 2.122 | 1.7229999999999994 | 1.342 | 1.0249999999999992 | 0.813 | 0.3420000000000001 | 2.1109999999999998 |
| 560 | 1.822 | 1.592 | 1.1819999999999993 | 0.8120000000000004 | 0.528 | 0.21800000000000008 | 1.816 |
| 580 | 1.623 | 1.4129999999999994 | 1.0009999999999992 | 0.517 | 0.33800000000000024 | 0.161 | 1.614 |
| 600 | 1.4059999999999986 | 1.125 | 0.816 | 0.3190000000000002 | 0.201 | 0.12200000000000004 | 1.4 |(C)
(D)
### Chart
| Category | 0 | 25 | 50 | 100 | 150 | 200 | Solvent |
|---|---|---|---|---|---|---|---|
| 460 | 1.617 | 1.351 | 1.091 | 0.887 | 0.6420000000000003 | 0.201 | 1.602 |
| 480 | 1.951 | 1.772 | 1.49 | 1.171 | 0.882 | 0.37900000000000017 | 1.9419999999999993 |
| 500 | 2.316999999999999 | 2.023 | 1.728 | 1.381 | 1.073 | 0.527 | 2.304 |
| 520 | 2.3919999999999986 | 1.981 | 1.736 | 1.397 | 1.097 | 0.532 | 2.3749999999999987 |
| 540 | 2.222 | 1.823 | 1.542 | 1.165 | 0.8630000000000003 | 0.402 | 2.2163999999999997 |
| 560 | 1.9720000000000006 | 1.592 | 1.302 | 0.912 | 0.6280000000000003 | 0.31800000000000017 | 1.9620000000000006 |
| 580 | 1.7229999999999994 | 1.343 | 1.101 | 0.6070000000000003 | 0.43800000000000017 | 0.229 | 1.712 |
| 600 | 1.484 | 1.115 | 0.886 | 0.40900000000000014 | 0.31100000000000017 | 0.167 | 1.472 |
### Chart
| Category | 0 | 25 | 50 | 100 | 150 | 200 | Solvent |
|---|---|---|---|---|---|---|---|
| 460 | 1.687 | 1.5509999999999993 | 1.5109999999999992 | 1.187 | 0.7420000000000003 | 0.3040000000000002 | 1.6819999999999993 |
| 480 | 1.931 | 1.792 | 1.693 | 1.371 | 1.082 | 0.597 | 1.9319999999999993 |
| 500 | 2.356999999999999 | 2.123 | 1.9279999999999993 | 1.581 | 1.2829999999999993 | 0.7240000000000003 | 2.3419999999999987 |
| 520 | 2.3619999999999997 | 2.128 | 1.9359999999999993 | 1.597 | 1.2889999999999993 | 0.7290000000000003 | 2.352999999999999 |
| 540 | 2.242 | 2.0129999999999986 | 1.722 | 1.4049999999999994 | 1.103 | 0.586 | 2.231 |
| 560 | 2.1719999999999997 | 1.9920000000000007 | 1.502 | 1.212 | 0.9580000000000003 | 0.41500000000000015 | 2.1519999999999997 |
| 580 | 1.973 | 1.843 | 1.381 | 1.087 | 0.7380000000000003 | 0.31400000000000017 | 1.961 |
| 600 | 1.8839999999999992 | 1.7149999999999994 | 1.236 | 0.8590000000000003 | 0.541 | 0.224 | 1.8779999999999992 |(E)
Figure S3. DPPH radical scavenging activity of bare ZnO nanoparticles. Various concentrations (0, 25, 50, 100, 150, & 200 mg/mL) of bare ZnO nanoparticles were added to freshly prepare stable DPPH solution (6 x 10^-5^ M), mixed and incubated at room temperature for 60 min. Then, DPPH radical scavenging potential was monitored by UV-vis spectrophotometer in the range of A460−600 nm wavelength. DPPH radical scavenging activity was measured at (A) 3, (B) 6, (C) 9, (D) 12 and (E) 15 months of storage.

## Slide 4
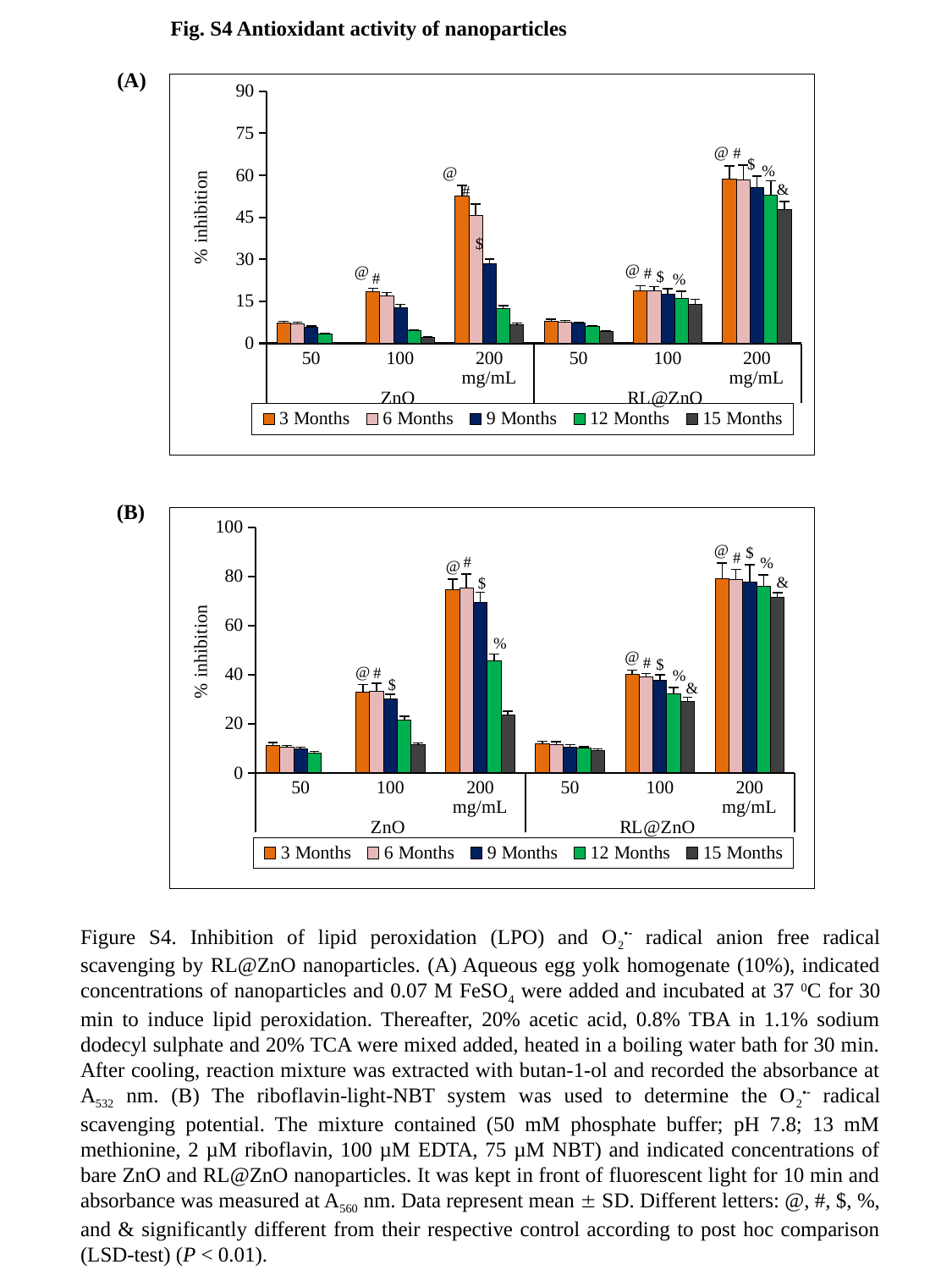

Fig. S4 Antioxidant activity of nanoparticles
(A)
### Chart
| Category | 3 Months | 6 Months | 9 Months | 12 Months | 15 Months |
|---|---|---|---|---|---|
| 50 | 7.31 | 7.02 | 5.6 | 3.2 | 0.0 |
| 100 | 18.5 | 16.9 | 12.7 | 4.41 | 2.17 |
| 200 mg/mL | 52.61 | 45.6 | 28.3 | 12.51 | 6.51 |
| 50 | 7.73 | 7.49 | 7.26 | 6.119999999999997 | 4.1499999999999995 |
| 100 | 18.7 | 18.8 | 17.4 | 16.1 | 13.9 |
| 200 mg/mL | 58.61 | 58.4 | 55.5 | 52.9 | 47.8 |@
#
$
%
@
&
#
$
@
@
#
$
#
%
(B)
### Chart
| Category | 3 Months | 6 Months | 9 Months | 12 Months | 15 Months |
|---|---|---|---|---|---|
| 50 | 11.229999999999999 | 10.4 | 9.729999999999999 | 8.120000000000001 | 0.0 |
| 100 | 32.76000000000001 | 33.21 | 30.2 | 21.34 | 11.34 |
| 200 mg/mL | 74.6 | 75.34 | 69.3 | 45.7 | 23.5 |
| 50 | 11.94 | 11.53 | 10.58 | 10.02 | 9.030000000000001 |
| 100 | 39.94 | 38.93 | 37.72000000000001 | 32.2 | 28.9 |
| 200 mg/mL | 79.2 | 78.61 | 77.7 | 75.83 | 71.49 |@
$
#
#
%
@
&
$
%
@
#
$
@
#
%
$
&
Figure S4. Inhibition of lipid peroxidation (LPO) and O2•− radical anion free radical scavenging by RL@ZnO nanoparticles. (A) Aqueous egg yolk homogenate (10%), indicated concentrations of nanoparticles and 0.07 M FeSO4 were added and incubated at 37 0C for 30 min to induce lipid peroxidation. Thereafter, 20% acetic acid, 0.8% TBA in 1.1% sodium dodecyl sulphate and 20% TCA were mixed added, heated in a boiling water bath for 30 min. After cooling, reaction mixture was extracted with butan-1-ol and recorded the absorbance at A532 nm. (B) The riboflavin-light-NBT system was used to determine the O2•− radical scavenging potential. The mixture contained (50 mM phosphate buffer; pH 7.8; 13 mM methionine, 2 µM riboflavin, 100 µM EDTA, 75 µM NBT) and indicated concentrations of bare ZnO and RL@ZnO nanoparticles. It was kept in front of fluorescent light for 10 min and absorbance was measured at A560 nm. Data represent mean ± SD. Different letters: @, #, $, %, and & significantly different from their respective control according to post hoc comparison (LSD-test) (P < 0.01).
